# Adult-onset citrullinaemia type II with liver cirrhosis: A rare cause of hyperammonaemia

**DOI:** 10.1515/med-2021-0235

**Published:** 2021-03-23

**Authors:** Pingrun Chen, Xin Gao, Bin Chen, Yan Zhang

**Affiliations:** Department of Gastroenterology, West China Hospital of Sichuan University, Chengdu, Sichuan, China

**Keywords:** hyperammonaemia, liver transplantation, adult-onset citrullinaemia type II

## Abstract

Adult-onset citrullinaemia type II (CTLN2) is a rare disease in Chinese patients. As a subtype of citrin deficiency (CD), it is an autosomal recessive disease related to the SLC25A13 mutation on chromosome 7q21.3. In this study, we report a case of CTLN2 presenting with paroxysmal altered consciousness and refractory hyperammonaemia. The diagnosis was finally confirmed by gene analysis. The patient recovered after liver transplantation. It can be learned from this case that CD should be considered in patients with refractory hyperammonaemia and paroxysmal mental disorder without a history of liver disease.

## 1 Introduction

Altered consciousness can be caused by many diseases such as epilepsy, schizophrenia, and metabolic encephalopathy. Metabolic encephalopathies are a group of disorders defined as brain malfunction secondary to systemic metabolic derangements. Hyperammonaemia is one of the aetiologies. As the liver is the main organ of ammonia metabolism, hyperammonaemia is often considered a common complication of chronic liver disease. In fact, any disease involved in the urea cycle can cause hyperammonaemia. Citrin, a liver-type mitochondrial aspartate–glutamate carrier, plays an important role in supplying aspartate to argininosuccinate synthetase in the cytosol to generate argininosuccinate in the urea cycle. Citrin deficiency (CD) can cause urea circulation disorder, which leads to hyperammonaemia. There are two kinds of CD, neonatal intrahepatic cholestasis (NICCD) and adult-onset citrullinaemia type II (CTLN2) [1]. In China, there have been several reports on NICCD [2,3], but few about CTLN2. Patients with CTLN2 display various neurological symptoms. It is on the list of rare diseases in China. In this study, we report a case of adult-onset citrullinaemia type II (CTLN2) in which the diagnosis was finally confirmed by gene analysis.

## 2 Case report

An 18-year-old man with paroxysmal altered consciousness for half a year was admitted to our hospital. The patient complained of episodic delirium, screaming, convulsion, and vomiting every 5–8 days, each time lasting for a few hours before the symptoms spontaneously alleviated. The patients did not provide any precipitating factors before the onset of these symptoms but confessed a preference for foods such as nuts, peanuts, eggs, and meat, as well as an aversion to rice or sweet desserts. The initial local hospital examinations revealed no abnormalities in the brain or abdominal computed tomography but a significant increase in plasma ammonia levels (296 μmol/L, reference range: 11–35 μmol/L), and a slight elevation of alanine aminotransferase (ALT, 79.16 U/L, reference range: 7–56 U/L) and aspartate aminotransferase (AST, 87.98 U/L, reference range: 10–40 U/L). Tests for EBV, CMV, HAV, HBV, HCV, HIV, thyroid function, ceruloplasmin, markers of autoimmune liver disease, and α-antitrypsin were negative. Data from another hospital 1 month prior also showed a significant increase in plasma ammonia levels (209 μmol/L), and a slight elevation of ALT (77 U/L) and AST (80.15 U/L) levels. Metabolic encephalopathy was diagnosed and treated. As the symptoms recurred with an increased frequency of paroxysmal altered consciousness, the patient was transferred to our hospital.

**Figure 1:**
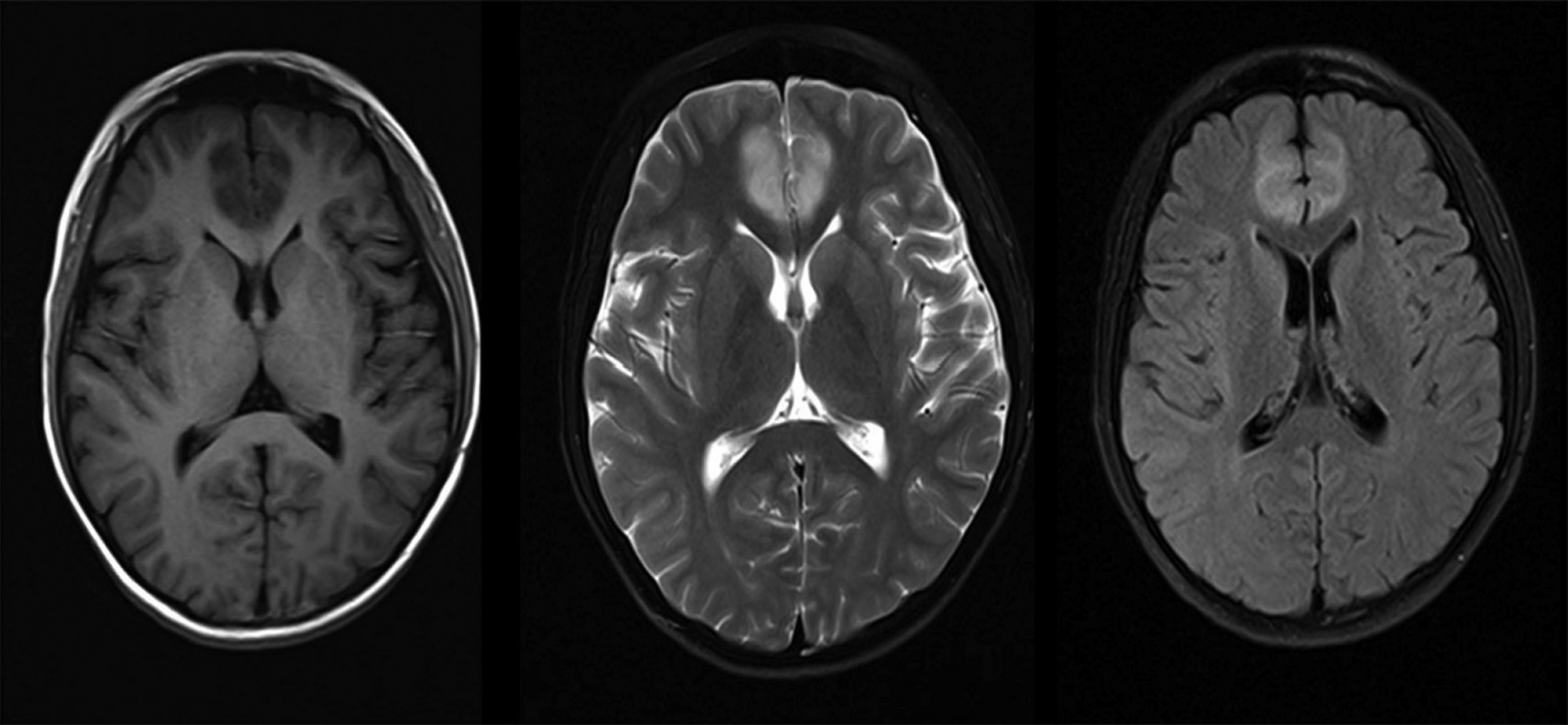
Brain MRI shows a region of high signal intensity in bilateral frontal lobe.

**Figure 2:**
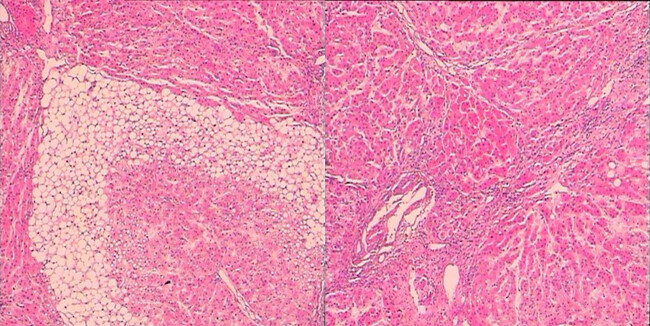
Histopathology of liver showed nodular cirrhosis with mixed-type steatosis involving approximately 60% of hepatocytes.

On admission, no abnormalities were found on physical examination. The levels of plasma ammonia (543.9 μmol/L), ALT (88 IU/L), and AST (149 IU/L) were still high. Despite the use of arginine and ornithine aspartate, the patient slipped into a coma without precipitating factors. Both pupils were equally round at approximately 2.5 mm each with a slow light reflex. Active tendon reflexes and positive Babinski signs were elicited and interpreted as the result of cerebral oedema. Mannitol and furosemidum were administered, and the patient recovered consciousness. Brain magnetic resonance imaging (MRI) showed a region of high signal intensity in the bilateral frontal lobes, which indicated the possibility of metabolic encephalopathy or infarction (Figure 1). Urine organic acid analysis revealed increased levels of lactic acid and pyruvate. The level of serum citrulline was also markedly elevated (519.256 µMm, normal <40 µMm). Liver pathology showed nodular cirrhosis with mixed-type steatosis involving approximately 60% of hepatocytes (Figure 2).

**Figure 3:**
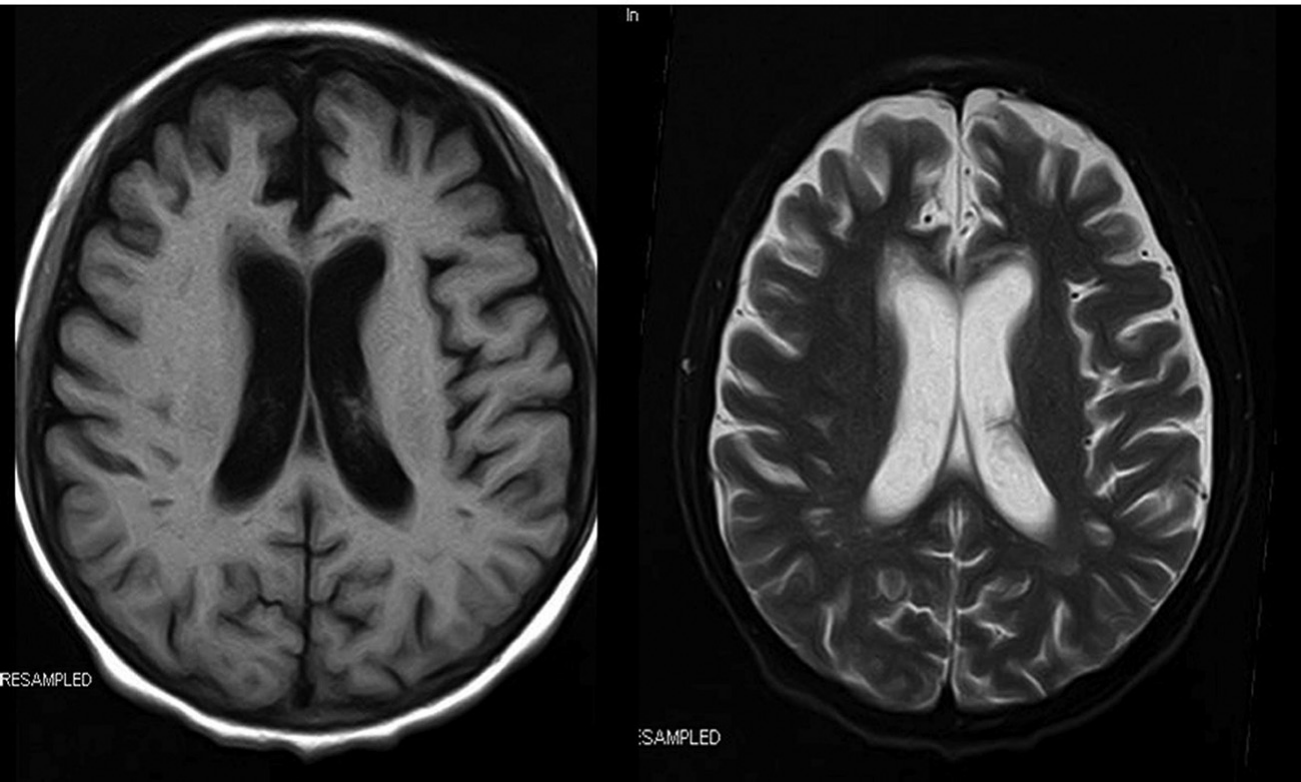
Brain MRI indicated cerebral atrophy after liver transplantation.

SLC25A13 gene analysis showed that the patient was a compound heterozygote for c.851-854del and IVS16ins3kb. His mother had a heterozygous mutation in c.851-854del. Adult-onset citrullinaemia type II (CTLN2) was diagnosed. After conservative treatment including arginine, ornithine aspartate, sodium pyruvate, glutathione, ursodeoxycholic acid, and a low-carbohydrate, high medium-chain triglycerides (MCT) and lactose-free diet, the patient recovered significantly. One month later, the patient received liver transplantation, after which his blood ammonia level dropped to 61 µmol/L. Approximately 1 month after the surgery, brain MRI indicated cerebral atrophy (Figure 3). Fortunately, after the 4-year follow-up, the patient recovered without recurrence.

Consent for the publication of this case has been obtained from the patient.

## 3 Discussion

CTLN2 is a rare disease in the Chinese population. As a subtype of CD, it is an autosomal recessive disease related to the SLC25A13 mutation on chromosome 7q21.3. Several genotypes of SLC25A13 have been discovered, such as c.851-854del, IVS16ins3kb, IVS6 + 5G > A, and c.1177 + 1G4A [3,4]. To date, the relationship between genotypes and clinical features in CTLN2 has not been illustrated. Some studies have demonstrated that there is no obvious relationship between genotypes and clinical outcomes in NICCD [5,6]. The estimated frequencies of SLC25A13 homozygotes reported by Lu et al. were one in 17,000 in the Chinese population, one in 19,000 in the Japanese population, and one in 50,000 in the Korean population [3,7]. To date, there have been no data from western populations. The most characteristic feature of CTLN2 is the late onset of symptoms, including delirium and abnormal behaviours, ranging from age 11 to 79 [8]. Many CD patients show a preference for protein/lipid-rich foods and an aversion to carbohydrates and sweets. A high-carbohydrate diet may trigger CTLN2, and discrepancies in CTLN2 incidence may be related to variations in food customs [8]. Because of the unique food preferences of CTLN2 patients, researchers have focused on diet therapy. Hayasaka demonstrated the efficacy of MCT supplementary therapy on CTLN2, and all patients in his study responded [9]. MCT can increase the nicotinamide adenine dinucleotide-oxidized (NAD^+^)/NADH ratio through the malate–citrate shuttle and can reduce oxidative stress [8]. Lactose can decrease the NAD/NADH ratio in the liver which may exacerbate the disease [8]. Naito described a patient whose condition was improved after lactose-free formula and deteriorated after lactose-containing formula [10].

Because of the non-specificity of symptoms, CTLN2 is easily misdiagnosed. Elevation of the plasma ammonia concentration is common in CTLN2. Serving as an aspartate–glutamate transporter in the mitochondria, citrin is essential for the urea cycle and NADH shuttle [8]. CD leads to citrulline metabolic disturbances and urea circulation disorder, which results in elevated plasma ammonia levels and metabolic encephalopathy. The final diagnosis of CTLN2 depends on genetic testing. To date, liver transplantation is the only definitive treatment for patients with CTLN2. Kimura et al. showed that 29 of the 56 (51.8%) patients in the conservative treatment group died within a few years of onset, while the 21 patients who received liver transplantation all survived [11]. According to the Japanese Liver Transplantation Society, 52 citrullinaemia patients had received liver transplantation from 1992 to the end of 2013, and the cumulative 10-year survival rate after surgery was up to 96.1% [12].

In our case, the patient was very young with a short disease course and rapid progression. Liver cirrhosis and brain atrophy are usually seen in a long disease course. In addition, it is uncommon to find hepatic cirrhosis in a young man with a short duration of CTLN2. Chen reported a case of CTLN2 with a course of 6 months. Brain MRI revealed bilateral symmetric lesions of the cingulate cortex, which disappeared after liver transplantation [13].

## 4 Conclusion

In conclusion, our case indicated that CTLN2 should be considered in patients who present with paroxysmal altered consciousness and refractory hyperammonaemia. Unusual food preferences may provide some clues for the diagnosis. Some conservative treatments may induce a temporary remission of hyperammonaemia and altered consciousness but failed to achieve long-term improvement, and most of the patients finally died of severe brain damage [11]. Therefore, early diagnosis and timely transfer of these patients to liver transplantation are of utmost importance.

## References

[1] Kikuchi A, Arai-Ichinoi N, Sakamoto O, Matsubara Y, Saheki T, Kobayashi K, et al. Simple and rapid genetic testing for citrin deficiency by screening 11 prevalent mutations in SLC25A13. Mol Genet Metab. 2012;105(4):553–8. 10.1016/j.ymgme.2011.12.024 22277121

[2] Song YZ, Zhang ZH, Lin WX, Zhao XJ, Deng M, Ma YL, et al. SLC25A13 gene analysis in citrin deficiency: sixteen novel mutations in East Asian patients, and the mutation distribution in a large pediatric cohort in China. PLoS One. 2013;8(9):e74544. 10.1371/journal.pone.0074544 PMC3777997 24069319

[3] Lin WX, Zeng HS, Zhang ZH, Mao M, Zheng QQ, Zhao ST, et al. Molecular diagnosis of pediatric patients with citrin deficiency in China: SLC25A13 mutation spectrum and the geographic distribution. Sci Rep. 2016;6:29732. 10.1038/srep29732 PMC4942605 27405544

[4] Oh SH, Lee BH, Kim GH, Choi JH, Kim KM, Yoo HW. Biochemical and molecular characteristics of citrin deficiency in Korean children. J Hum Genet. 2017;62(2):305–7. 10.1038/jhg.2016.131 27829683

[5] Xing YZ, Qiu WJ, Ye J, Han LS, Xu SS, Zhang HW, et al. Studies on the clinical manifestation and SLC25A13 gene mutation of Chinese patients with neonatal intrahepatic cholestasis caused by citrin deficiency. Zhonghua Yi Xue Yi Chuan Xue Za Zhi. 2010;27(2):180–5. 10.3760/cma.j.issn.1003-9406.2010.02.014 20376801

[6] Song YZ, Deng M, Chen FP, Wen F, Guo L, Cao SL, et al. Genotypic and phenotypic features of citrin deficiency: five-year experience in a Chinese pediatric center. Int J Mol Med. 2011;28(1):33–40. 10.3892/ijmm.2011.653 21424115

[7] Lu YB, Kobayashi K, Ushikai M, Tabata A, Iijima M, Li MX, et al. Frequency and distribution in East Asia of 12 mutations identified in the SLC25A13 gene of Japanese patients with citrin deficiency. J Hum Genet. 2005;50(7):338–46. 10.1007/s10038-005-0262-8 16059747

[8] Hayasaka K, Numakura C. Adult-onset type II citrullinemia: current insights and therapy. Appl Clin Genet. 2018;11:163–70. 10.2147/TACG.S162084 PMC6296197 30588060

[9] Hayasaka K, Numakura C, Yamakawa M, Mitsui T, Watanabe H, Haga H, et al. Medium-chain triglycerides supplement therapy with a low-carbohydrate formula can supply energy and enhance ammonia detoxification in the hepatocytes of patients with adult-onset type II citrullinemia. J Inherit Metab Dis. 2018;41(5):777–84. 10.1007/s10545-018-0176-1 29651749

[10] Naito E, Ito M, Matsuura S, Yokota, Saijo T, Ogawa Y, et al. Type II citrullinaemia (citrin deficiency) in a neonate with hypergalactosaemia detected by mass screening. J Inherit Metab Dis. 2002;25(1):71–6. 10.1023/a:1015198103395 11999983

[11] Kimura N, Kubo N, Narumi S, Toyoki Y, Ishido K, Kudo D, et al. Liver transplantation versus conservative treatment for adult-onset type II citrullinemia: our experience and a review of the literature. Transplant Proc. 2013;45(9):3432–7. 10.1016/j.transproceed.2013.06.016 24182831

[12] Umeshita K, Inomata Y, Furukawa H, Kasahara M, Kawasaki S, Kobayashi E, et al. Liver transplantation in Japan: registry by the Japanese liver transplantation. Soc Hepatol Res Off J Jpn Soc Hepatol. 2016;46(12):1171–86. 10.1111/hepr.12676 26887781

[13] Chen L, Zhao B, Shang H. Teaching neuroimages: reversible brain MRI lesions in adult-onset type II citrullinemia. Neurology. 2017;89(9):e115. 10.1212/WNL.0000000000004298 28847840

